# ZIKV induction of tristetraprolin in endothelial and Sertoli cells post-transcriptionally inhibits IFNβ/λ expression and promotes ZIKV persistence

**DOI:** 10.1128/mbio.01742-23

**Published:** 2023-09-14

**Authors:** William R. Schutt, Jonas N. Conde, Megan C. Mladinich, Grace E. Himmler, Erich R. Mackow

**Affiliations:** 1 Department of Microbiology and Immunology, Stony Brook University, Stony Brook, New York, USA; 2 Center for Infectious Disease, Stony Brook University, Stony Brook, New York, USA; 3 Molecular and Cell Biology Program, Stony Brook University, Stony Brook, New York, USA; Indiana University Bloomington, Bloomington, Indiana, USA

**Keywords:** Zika virus, tristetraprolin, endothelial cells, Sertoli cells, interferon regulation, persistence, post-transcriptional, secretion

## Abstract

Zika virus (ZIKV) is a mosquito-borne *Flavivirus* that persistently infects patients; enters protected brain, placental, and testicular compartments; is sexually transmitted; and causes fetal microcephaly *in utero*. ZIKV persistently infects human brain microvascular endothelial cells (hBMECs) that form the blood-brain barrier and Sertoli cells that form testicular barriers, establishing reservoirs that enable viral dissemination. ZIKV persistence requires inhibiting interferon (IFN) responses that direct viral clearance. We found that ZIKV induces IFNβ and IFNλ in hBMECs but post-transcriptionally inhibits IFNβ/IFNλ expression. IFNβ/IFNλ mRNAs contain AU-rich elements (AREs) in their 3′ untranslated regions which regulate protein expression through interactions with ARE-binding proteins (ARE-BPs). We found that ZIKV infection of primary hBMECs induces the expression of the ARE-BP tristetraprolin (TTP) and that TTP is a novel regulator of endothelial IFN secretion. In hBMECs, TTP knockout (KO) increased IFNβ/IFNλ mRNA abundance and IFNβ/IFNλ secretion in response to ZIKV infection and inhibited viral persistence. In contrast, TTP expression dramatically reduced IFNβ/IFNλ secretion in hBMECs. IFNβ/IFNλ mRNA stability was not significantly altered by TTP and is consistent with TTP inhibition of IFNβ/IFNλ translation. TTP is similarly induced by ZIKV infection of Sertoli cells, and like hBMECs, TTP expression or KO inhibited or enhanced IFNβ/IFNλ mRNA levels, respectively. These findings reveal a mechanism for ZIKV-induced TTP to promote viral persistence in hBMECs and Sertoli cells by post-transcriptionally regulating IFNβ/IFNλ secretion. Our results demonstrate a novel role for virally induced TTP in regulating IFN secretion in barrier cells that normally restrict viral persistence and spread to protected compartments.

**IMPORTANCE:**

Our findings define a novel role for ZIKV-induced TTP expression in regulating IFNβ/IFNλ production in primary hBMECs and Sertoli cells. These cells comprise key physiological barriers subverted by ZIKV to access brain and testicular compartments and serve as reservoirs for persistent replication and dissemination. We demonstrate for the first time that the ARE-binding protein TTP is virally induced and post-transcriptionally regulates IFNβ/IFNλ secretion. In ZIKV-infected hBMEC and Sertoli cells, TTP knockout increased IFNβ/IFNλ secretion, while TTP expression blocked IFNβ/IFNλ secretion. The TTP-directed blockade of IFN secretion permits ZIKV spread and persistence in hBMECs and Sertoli cells and may similarly augment ZIKV spread across IFNλ-protected placental barriers. Our work highlights the importance of post-transcriptional ZIKV regulation of IFN expression and secretion in cells that regulate viral access to protected compartments and defines a novel mechanism of ZIKV-regulated IFN responses which may facilitate neurovirulence and sexual transmission.

## INTRODUCTION

Zika virus (ZIKV) is a mosquito-borne neurovirulent *Flavivirus* that uniquely spreads sexually, persists for months in humans, and causes encephalitis and fetal microcephaly *in utero* (1 – 5). In adults, ZIKV is found persistently in saliva, urine, cerebrospinal fluid, and semen causing encephalitis, Guillian-Barre syndrome, and permitting sexual transmission (5 – 7). During pregnancy, ZIKV spreads across the placenta and establishes persistence in Hofbauer cells, fetal macrophages that serve as reservoirs for ZIKV fetal dissemination in infected humans and remain infected regardless of the gestational term at which initial infection occurred (8, 9). Subsequent viral spread and infection of the developing brain result in damage to fetal neurons, neural progenitors, and astrocytes that limit brain development and can result in characteristic fetal microcephaly (10, 11). Accordingly, ZIKV infects and crosses endothelial cell (EC), placental, and testicular barriers that normally restrict viral access to the central nervous system (CNS), fetal tissues, and semen (1, 7, 12 – 14). The blood-brain barrier (BBB) is a neurovascular complex formed by brain microvascular endothelial cells (BMECs), pericytes, and astrocytes that separate the brain from circulating blood constituents and pathogens. Our lab established that ZIKV persistently and non-lytically infects human brain microvascular endothelial cells (hBMECs), releasing progeny virus both apically and basolaterally without permeabilizing model BBBs (15 – 17). Additional studies have shown that Sertoli cells that form testicular barriers are persistently infected by ZIKV (18, 19). These findings suggest roles for hBMECs and Sertoli cells as ZIKV reservoirs that facilitate neuroinvasion and sexual transmission.

ECs are a unique cell type with distinct receptors, signaling pathways, and interferon (IFN)-directed responses that differentiate them from immune and epithelial cells and tailor their response to viral infection (20, 21). The endothelium is a target for many tick- and mosquito-borne flaviviruses that cause vascular or neurotropic diseases, including dengue virus (DENV), West Nile virus, and Powassan virus (22 – 24). ECs produce type I IFNβ and type III IFNλ in response to viral infections and respond to IFNα/IFNβ through cognate type I IFN receptors (IFNARs). ECs lack IFNλ receptors (IFNλRs), and the addition of IFNλ to cells fails to inhibit ZIKV infection or induce interferon-stimulated genes (ISGs). Prior treatment of cells with IFNα/IFNβ inhibits ZIKV and other *Flavivirus* infections, and DENV infection of human ECs induces IFNα/IFNβ secretion that inhibits DENV spread and ultimately leads to viral clearance (15, 24 – 26). In contrast, ZIKV persistently infects hBMECs without IFNβ secretion, spreading in monolayers for >9 days and following cellular passage (15, 17). Similar ZIKV persistence has been reported in Sertoli cells, an epithelial cell type that comprises the blood-testes barrier and is hypothesized to facilitate viral entry into the testes and contribute to long-term viral shedding and sexual transmission (18, 19, 27). How ZIKV persists within brain and testicular barrier cells is not fully understood; however, the ability of ZIKV to restrict IFN responses that prevent viral clearance is likely central to ZIKVs novel persistence, spread, and neurovirulence.

ZIKV has multiple mechanisms of inhibiting IFN responses both upstream and downstream of IFN induction and secretion (28). To impair viral RNA recognition, ZIKV inhibits the activation of retinoic acid-inducible gene I (RIG-I) and melanoma differentiation-associated protein 5 (MDA-5) via expression of several non-structural proteins and genomic sfRNA (29 – 34). ZIKV also inhibits IFN signaling directed by IFN receptor activation by inhibiting JAK-STAT2 signaling responses (25, 28, 35, 36). Despite suppression of the IFN induction and IFN signaling pathways, IFNβ and IFNλ are induced by ZIKV infection of a variety of cell types including hBMECs and Sertoli cells (15, 37, 38). Although IFN was induced in ZIKV-infected hBMECs, we found that the expression and secretion of IFNβ/IFNλ in cell supernatants were repressed below the limit of detection and failed to inhibit viral infection or spread (15). Secretory pathways were not globally altered by ZIKV as CCL5, a highly induced, pro-survival chemokine, was highly secreted into ZIKV-infected hBMEC supernatants. While most studies evaluate IFN transcripts as surrogates for IFN expression, a discrepancy between IFNβ/IFNλ induction and secretion has also been reported following ZIKV infection of dendritic cells, peripheral blood mononuclear cells, fetal neural progenitor cells, and placental macrophages (9, 37, 39, 40). A mechanism for post-transcriptional regulation of IFN has not been proposed, and the absence of IFN secretion was hypothesized to result from inhibited IFN translation (37).

IFNβ/IFNλ belong to a family of tightly regulated inflammatory cytokines, present at low endogenous levels, that undergo post-transcriptional regulation to rapidly repress secretion (41). Transcript regulation is reportedly mediated by the presence of AU-rich elements (AREs) found in 3′ untranslated regions (UTRs) of cytokine mRNAs. ARE recognition sequences drive interactions with ARE-binding proteins (ARE-BPs) that positively or negatively regulate mRNA translation and stability (41 – 48). How AREs and ARE-BPs influence IFNβ/IFNλ expression during ZIKV infection has not been investigated.

Studies involving the ARE-BP tristetraprolin (TTP) have largely focused on its role in lipopolysaccharide (LPS)-treated myeloid cells, with little understanding of its role in type I/type III IFN regulation or following viral infection. In ECs, expression of the TTP has been shown to regulate the expression of inflammatory cytokines (49, 50). TTP post-transcriptionally inhibits the expression of ARE-containing mRNAs through two separate mechanisms that may function independently or simultaneously (51). TTP can promote the degradation of ARE-mRNAs through deadenylation via recruitment of the CCR4-NOT exonuclease complexes or DCP1/2 decapping enzymes and Xrn1 digestion (52 – 57). TTP is also capable of translational repression via recruitment of inhibitory translational complexes to mRNAs that interfere with cap binding to eIF4E and translation initiation complex assembly (51, 58 – 61). How AREs and ARE-BPs influence IFNβ/IFNλ expression during ZIKV infection has not been studied.

Here we investigate ZIKV-directed post-transcriptional regulation of IFN expression and define roles for ARE-binding proteins in ZIKV persistence in hBMECs and Sertoli cells. In contrast to ARE-BPs AUF1, HuR, and KHSRP, we found that TTP is uniquely induced in ZIKV-infected hBMECs and that knockout (KO) or expression of TTP, respectively, increases or blocks IFNβ/IFNλ secretion. Our findings identify TTP as a ZIKV-induced protein in hBMECs and Sertoli cells that regulates the expression of IFNβ and IFNλ by inhibiting their translation. In addition, we found that ZIKV induces TTP in primary human Sertoli cells (hSerC) and that TTP expression or KO regulates IFNβ/IFNλ secretion. These findings reveal a post-transcriptional mechanism of ZIKV-directed IFN regulation resulting from TTP induction following infection and permit ZIKV persistence in cells that normally restrict viral entry into protected compartments.

## RESULTS

### ZIKV inhibits IFN secretion from hBMECs

**FIG 1:**
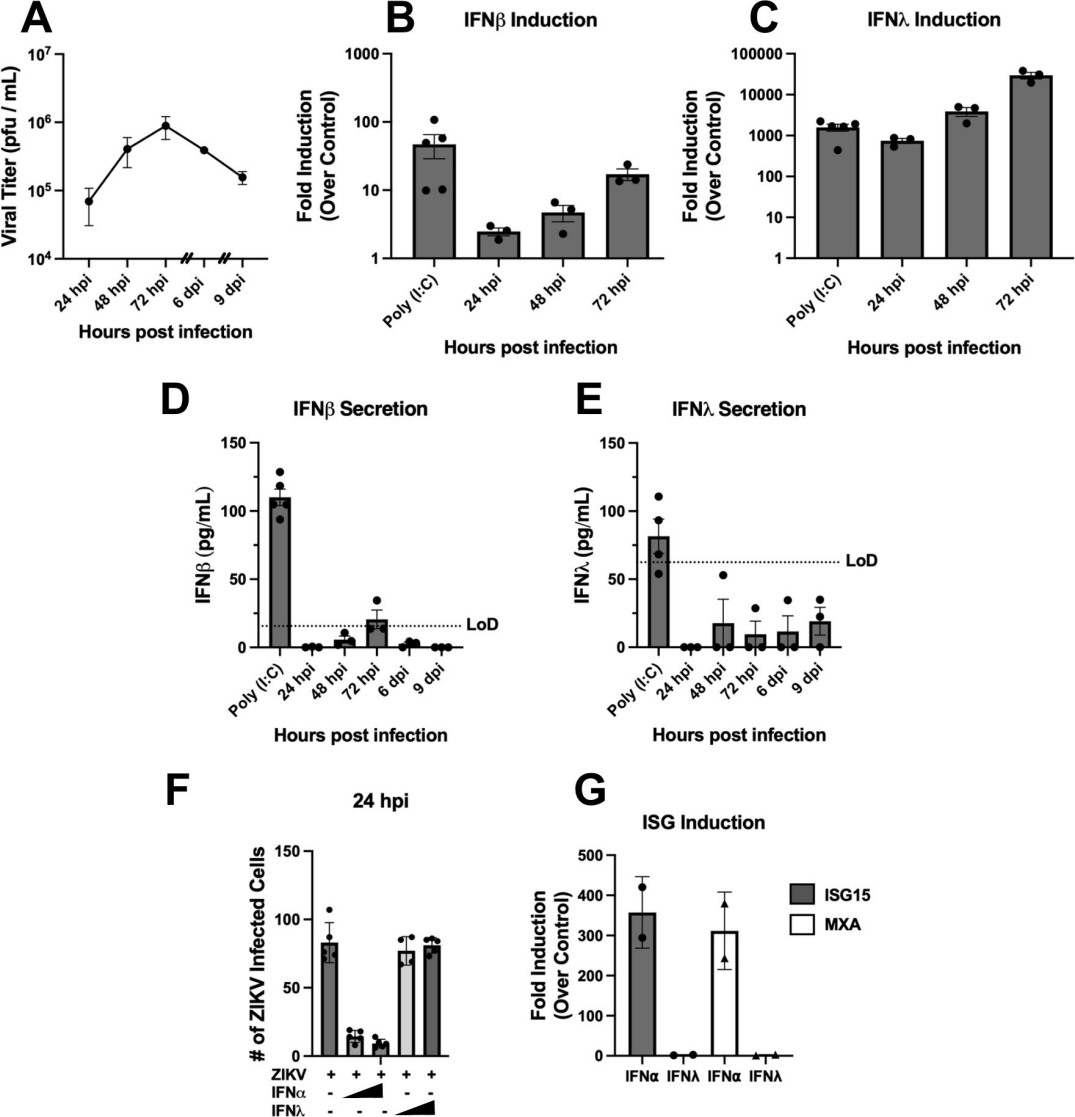
ZIKV Inhibits IFN secretion in in hBMECs. (**A**) Primary hBMECs were ZIKV infected (multiplicity of infection [MOI] = 0.05) and supernatants collected at the indicated times. Hash marks indicate when infected hBMECs were trypsinized and passaged (3 and 6 dpi). Viral titers were determined by focus forming units (FFU) assay on Vero E6 cells. (B through E) RNA and supernatants from ZIKV-infected hBMECs (MOI 0.05) were collected. The induction of IFNβ (**B**) and IFNλ (**C**) relative to uninfected control hBMECs was quantified via qRT-PCR and secreted IFNβ (**D**) and IFNλ (**E**) determined by ELISA. (**F and G**) Wild-type (WT) hBMECs were ZIKV infected (MOI 0.5) with or without IFNα (1,000 or 2,000 U/mL) or IFNλ_1_ (10 or 50 ng/mL) addition. The number of ZIKV antigen-positive hBMECs was quantified for 24 hpi using anti-DENV4 hyperimmune mouse ascitic fluid (**F**) and assessed for ISG induction relative to untreated controls (*n* = 2) (**G**). Data are represented as the mean ± standard error of the mean (SEM). Experiments were performed at least three times unless otherwise noted.

We previously reported that ZIKV persistently infects hBMECs, transiently inducing IFNβ and IFNβ/IFNλ without IFNβ/λ secretion (15). Here we extend these findings and define roles for the ARE-BP TTP in ZIKV-directed post-transcriptional regulation of IFNβ/IFNλ in hBMECs. We infected primary hBMECs with ZIKV (PRVABC59) and monitored viral spread and the induction and secretion of IFNβ and IFNλ. ZIKV titers increased from 24 to 72 hpi, and monolayers remained persistently infected despite passaging ZIKV-infected cells 3 and 6 dpi (Fig. 1A). Following ZIKV infection of hBMECs, we found that both IFNβ and IFNλ_1_ were transcriptionally induced 1–3 dpi (Fig. 1B and C), but we failed to detect IFNβ or IFNλ secretion into cell supernatants by enzyme-linked immunosorbent assay (ELISA) (Fig. 1D and E). In contrast, hBMECs transfected with poly(I:C) for 24 h directed the secretion of both IFNβ/IFNλ_1_ (Fig. 1D and E). Concurrently treating hBMECs with IFNα during ZIKV infection reduced the number of infected cells by 80%, while IFNλ-treated hBMECs had no effect on ZIKV infection (Fig. 1F). Consistent with this, ZIKV persistence and spread in hBMECs functionally demonstrate the absence of IFNβ secretion in ZIKV-infected hBMEC supernatants. Validating the presence of IFNα/IFNβ, but not IFNλ, receptors on hBMECs, cellular ISGs (ISG15 and MXA) were transcriptionally induced by IFNα but not by IFNλ addition to hBMECs (Fig. 1G). In contrast, treatment with both IFNα and IFNλ induce ISGs in A549 cells (Fig. S1), demonstrating that hBMECs only express type I IFNARs and selectively respond to IFNα/IFNβ. Our findings indicate that ZIKV infection of hBMECs post-transcriptionally inhibits IFNβ/IFNλ expression to facilitate ZIKV persistence and spread.

### The ARE-BP TTP is induced by ZIKV infection and localized to the cytoplasm

**FIG 2:**
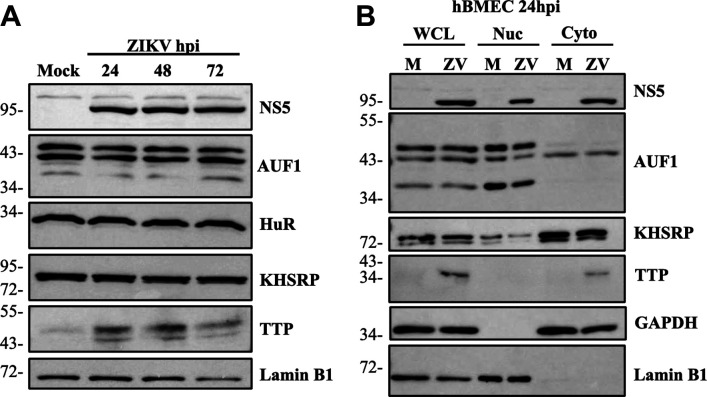
ZIKV induces TTP in the cytoplasm of hBMEC infection. (**A**) Primary hBMECs were ZIKV infected (MOI 1.0), and lysates were evaluated for the expression of ARE-BPs assessed by Western blot. (**B**) Mock and ZIKV-infected hBMECs (MOI 1.0) were lysed, and whole cell lysate was collected prior to centrifugation and splitting of cytoplasmic (Cyto) and nuclear (Nuc) fractions. Lysates were Western blotted for cytoplasmic glyceraldehyde 3-phosphate dehydrogenase (GAPDH) and nuclear lamin B1 protein markers to confirm localization and equal loading. Western blots are representative of multiple independent experiments.

It is widely reported that ZIKV infection transcriptionally induces IFNβ/IFNλ. To explain the discrepancy between IFN mRNA levels and a lack of protein secretion, we hypothesized that ZIKV regulates IFN expression and secretion at a post-transcriptional level. We first performed a miRNA screen of ZIKV-infected hBMECs at 24 hpi but failed to detect significant upregulation of any canonical IFNβ/IFNλ targeting miRNAs (data not shown). Based on the potential roles of AREs in cytokine 3′ UTRs regulating mRNA expression, we evaluated the expression of ARE-BPs in response to ZIKV infection of hBMECs. IFNβ and IFNλ contain 3′ UTR ARE domains that may regulate IFN expression in a cell type specific manner, and thus these experiments were performed in biologically relevant hBMECs in which post-transcriptional regulation has been observed (41, 42, 48). We evaluated expression levels of ARE-BPs HuR, KHSRP, AUF1, and TTP in ZIKV-infected hBMECs 1–3 dpi. We found that AUF1, HuR, and KHSRP were expressed constitutively in mock and ZIKV-infected hBMECs, with no change in expression following ZIKV infection (Fig. 2A). In contrast, TTP is expressed at extremely low constitutive levels in hBMECs and highly induced by ZIKV 1–3 dpi compared to mock-infected cells.

Regulation of mRNAs by ARE-BPs is linked to protein activation that directs localization from the nucleus to the cytoplasm (62 – 65). We evaluated ARE-BP localization in ZIKV versus mock-infected cells for 24 hpi and observed no change in the nuclear or cytoplasmic localization of AUF1 or KHSRP in mock- or ZIKV-infected hBMECs (Fig. 2B). The absence of TTP in mock-infected cells prevents analysis of a change in TTP localization, but the exclusive localization of TTP in the cytoplasm of ZIKV-infected hBMECs indicates the unique induction of activated TTP in ZIKV-infected hBMECs (Fig. 2B).

### ZIKV induction of TTP is independent of IRF3 and IFN

**FIG 3:**
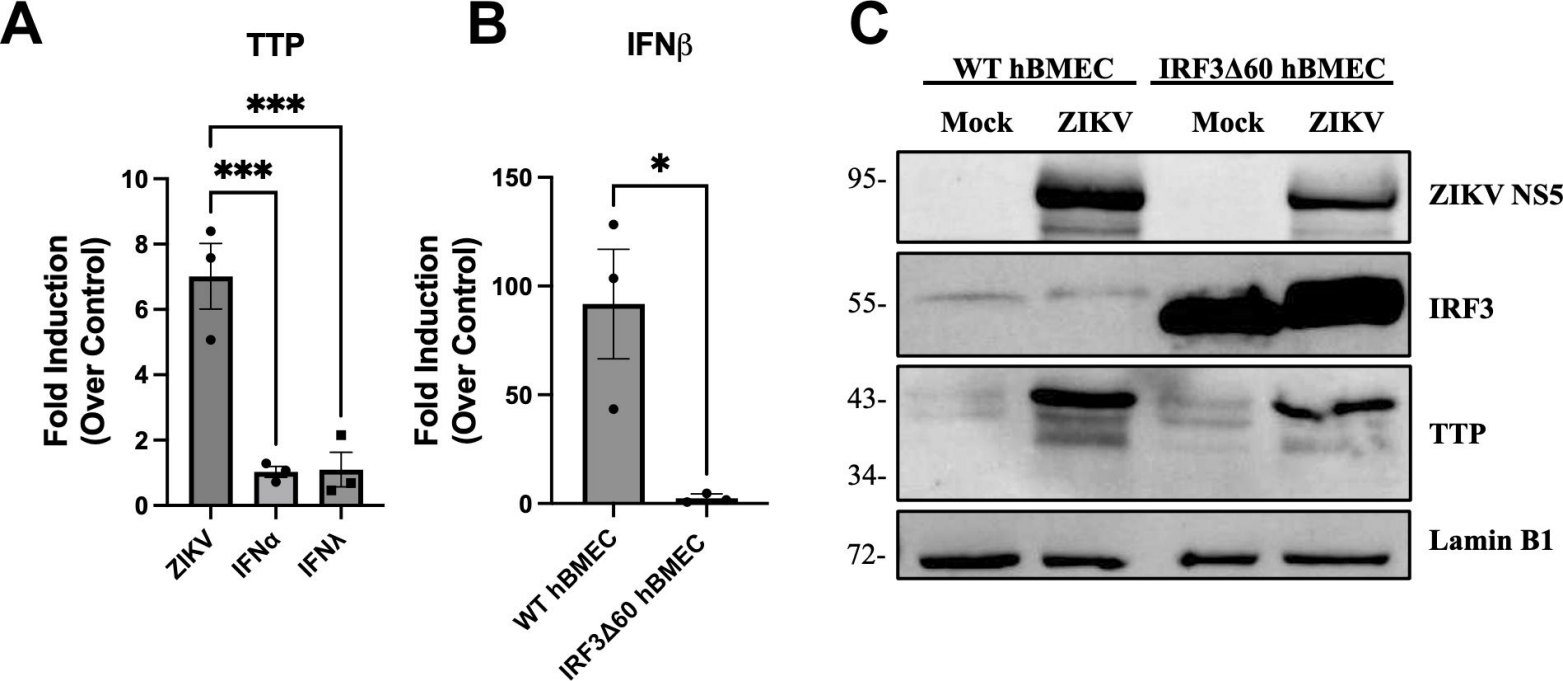
ZIKV induction of TTP is independent of IRF3 and IFN. (**A**) Primary hBMECs were ZIKV infected (MOI 10) and treated with exogenous IFNα (1,000 U/mL) or IFNλ (50 ng/mL) for 24 hpi prior to analysis of TTP transcripts by qRT-PCR relative to untreated control hBMECs. Statistical significance was determined by one-way analysis of variance (ANOVA). (**B and C**) Primary hBMEC and hBMECs expressing dominant negative IRF3Δ60 were ZIKV infected (MOI 10), and the transcriptional induction of IFNβ was determined by qRT-PCR relative to uninfected WT or IRF3Δ60-hBMECs (**B**) with significance defined by unpaired Student’s *t* test. Expression of IRF3 and TTP were analyzed by Western blot (**C**). Data are represented as the mean ± SEM; asterisks indicate statistical significance. *P ≤ 0.05, ***P ≤ 0.001. Experiments were performed three times.

TTP induction has primarily been studied in myeloid cells in response to inflammatory LPS and tumor necrosis factor alpha (TNF-α) stimuli, while the role of IFNs is unclear (62, 66). As IFNβ/IFNλ secretion is restricted in ZIKV-infected hBMECs, we compared TTP induction in response to ZIKV infection or stimulation with IFNα/IFNλ. hBMECs were ZIKV infected, treated with 1,000 U/mL of IFNα or 50 ng/mL of IFNλ for 24 h prior to analysis of TTP induction by qRT-PCR (Fig. 3A). We found that ZIKV virus infection, but not IFNα/IFNλ treatment, induced TTP in hBMECs. These findings are consistent with a previous study indicating that knockdown of RIG-I partially reduced TTP induction in response to RNA transfection (67). To determine if TTP induction is mediated by IRF3 activation following ZIKV infection, we transduced hBMECs to express a dominant negative IRF3 (IRF3Δ60) that lacks the ability to induce IFNβ (68). In response to ZIKV infection, IFNβ was induced in WT hBMECs but not IRF3Δ60-hBMECs, and IRF3Δ60 expression dramatically reduced IRF3-directed ISG expression (Fig. 3B; Fig. S2). Protein analysis revealed that TTP expression was induced by ZIKV infection of both WT and IRF3Δ60 hBMECs (Fig. 3C). These findings demonstrate that ZIKV infection of endothelial cells transcriptionally induces TTP expression independently of IRF3 or IFN signaling responses.

### TTP expression inhibits IFNβ/IFNλ expression

**FIG 4:**
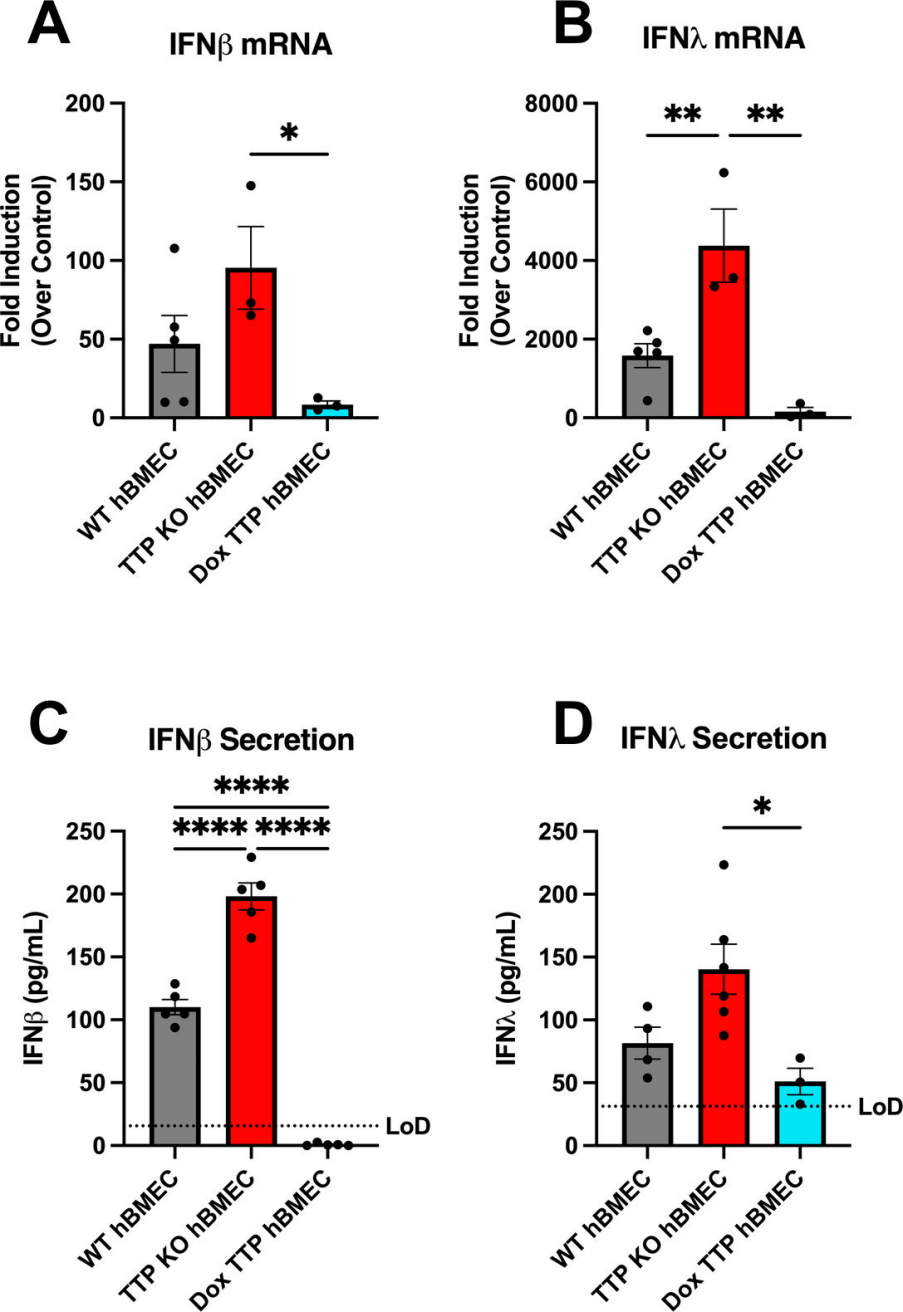
TTP expression inhibits IFNβ/IFNλ expression. Primary hBMECs, TTP-KO hBMECs, and TTP-expressing hBMECs were poly (I:C) transfected (0.5 µg/mL) using FuGENE 6 for 24 h prior to the collection of RNA and supernatants. TTP was doxycycline (Dox) induced (200 ng/mL) throughout the course of the experiment. Transcriptional induction of IFNβ (**A**) and IFNλ_1_ (**B**) was determined by qRT-PCR and normalized to untreated, uninfected WT, TTP KO, and Dox TTP controls. hBMEC supernatants were assayed for IFNβ (**C**) and IFNλ (**D**) by ELISA. Data are presented as the mean ± SEM; asterisks indicate statistical significance as determined by one-way ANOVA. *P ≤ 0.05, **P ≤ 0.001, **P ≤ 0.0001. Experiments were performed at least three times.

While TTP has been shown to inhibit the expression of some ARE-containing cytokines, regulation of IFNs remains understudied and has only been addressed in immune cells. To evaluate the effect of TTP on IFNβ/IFNλ induction and secretion in hBMECs, we generated TTP CRISPR-Cas9 KO and doxycycline-induced TTP overexpressing hBMECs (Fig. S3). Following poly(I:C) transfection, TTP KO hBMECs exhibited higher induction of IFNβ/IFNλ that was dramatically reduced by TTP overexpression (Fig. 4A and B). The enhanced IFN mRNA abundance corresponded with a significant increase in IFNβ/IFNλ secretion by TTP KO cells 24 h post-transfection (Fig. 4C and D). These findings demonstrate direct and novel IFNβ/IFNλ regulation by TTP in endothelial cells and suggest a mechanism for virally induced TTP to modulate inflammatory responses of the endothelium.

### ZIKV persistence is suppressed in TTP KOs and enhanced in TTP-expressing hBMECs

**FIG 5:**
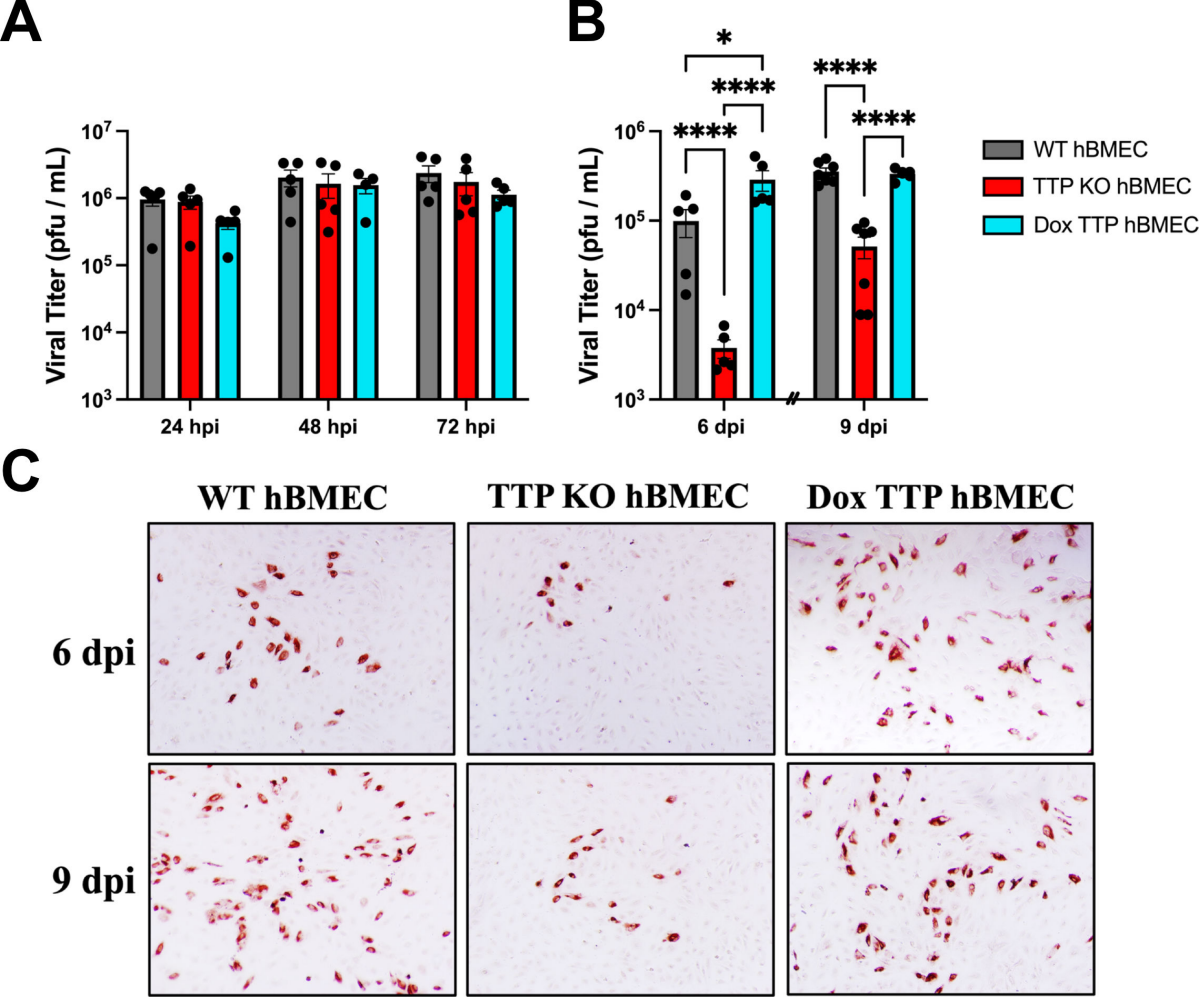
ZIKV persistence is suppressed in TTP KOs and enhanced in TTP-expressing hBMECs. (**A and B**) Primary hBMECs, TTP-KO hBMECs, and TTP-expressing hBMECs were ZIKV infected (MOI = 1), and supernatants were collected and titered on Vero E6 cells at the indicated times. (**B**) Infected hBMECs were trypsinized and passaged at 3 and 6 dpi (hash marks and titers quantified 3 days after passage). Data are presented as the mean ± SEM; asterisks indicate statistical significance as determined by two-way ANOVA of log10-transformed titers. *P ≤ 0.05, , ****P ≤ 0.0001). Experiments were performed four times. (**C**) Infected hBMECs were fixed, and ZIKV-positive cells wee stained with anti-DENV4 hyperimmune mouse ascitic fluid at 6 and 9 dpi.

We determined if TTP KO or TTP expression in hBMECs affected ZIKV replication and persistence in hBMECs. In a synchronous ZIKV infection of WT, TTP KO, and TTP-expressing hBMECs, we found that titers rapidly reached maximal levels (1 × 10^6^ /mL) 1–3 dpi with little difference between cell types (Fig. 5A). To assess roles for TTP in viral persistence, we passaged ZIKV-infected WT, TTP KO, and TTP-expressing cells at 3 and 6 dpi and monitored viral titers 6–9 dpi (Fig. 5B). We observed a significant reduction in persistently infected ZIKV titers in TTP KO hBMECs at 6 and 9 dpi versus hBMECs that express TTP (WT or TTP expressing). In support of TTP-regulated ZIKV persistence, staining of ZIKV-infected hBMECs 6–9 dpi revealed reduced ZIKV spread in TTP KO cells versus WT or TTP-expressing hBMECs (Fig. 5C). Collectively, these finding suggest that ZIKV-induced TTP expression suppresses IFN secretion and facilitates persistent viral spread.

**FIG 6:**
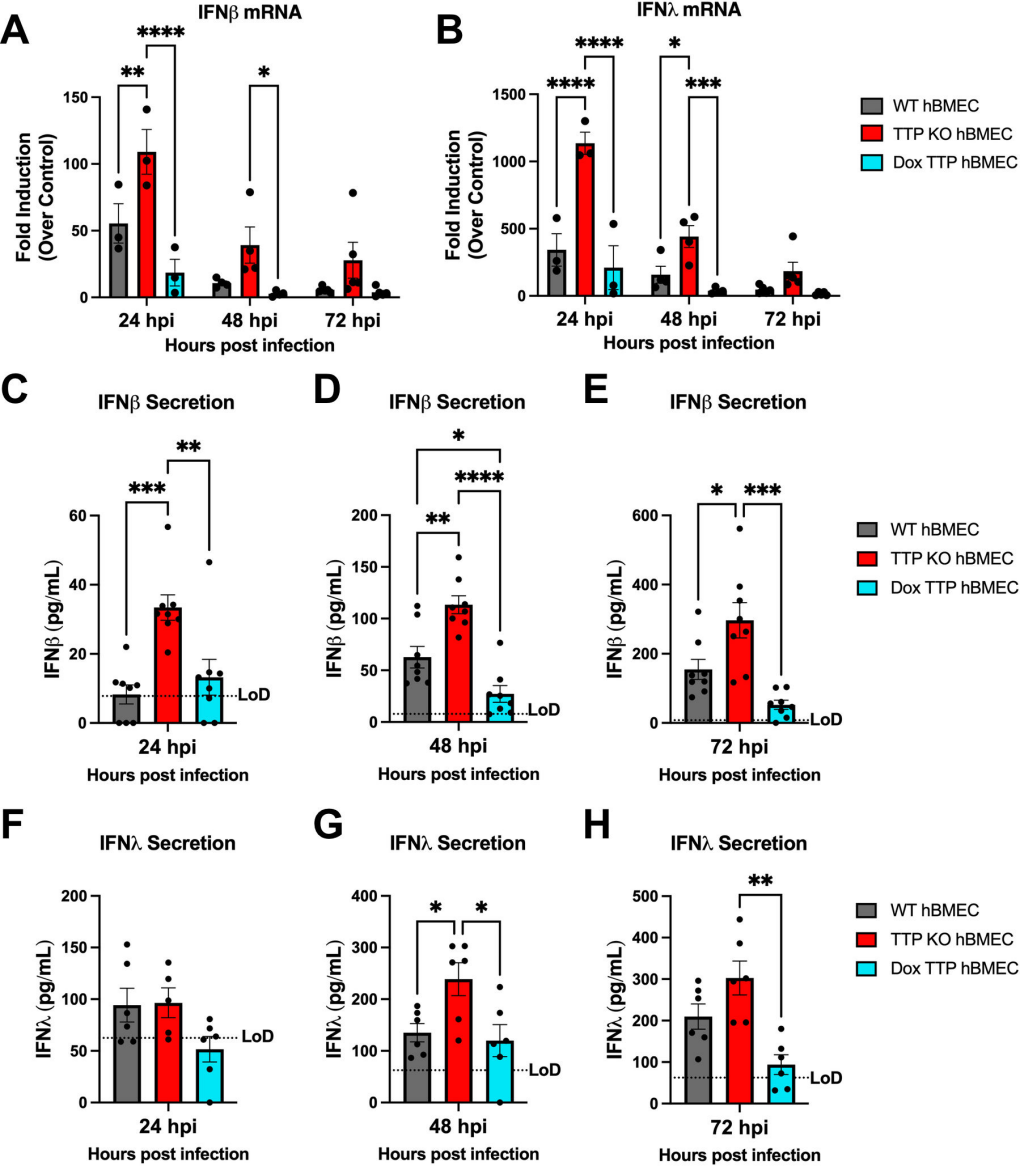
IFN induction and secretion is enhanced in ZIKV-infected TTP KO hBMECs. Primary hBMECs, TTP-KO hBMECs, and TTP-expressing hBMECs were ZIKV infected (MOI = 10), and at the indicated times, IFNβ (A) and IFNλ (B) mRNAs were quantified by qRT-PCR relative to uninfected WT, TTP KO, and Dox TTP controls, and secreted IFNβ (C through E) and IFNλ (F through H) were assayed by ELISA (R&D Systems). Data are presented as the mean ± SEM; asterisks indicate statistical significance as determined by two-way ANOVA. *P ≤ 0.05; **P ≤ 0.002, ***P ≤ 0.0002; ****P ≤ 0.0001. Experiments were performed four times.

We evaluated IFNβ/IFNλ induction and secretion in WT, TTP KO, and TTP-expressing hBMECs following synchronous (MOI = 10) ZIKV infection. In TTP KO cells, IFNβ/IFNλ_1_ was induced two- to fourfold over WT hBMECs 1–3 dpi (Fig. 6A and B). In contrast, TTP-expressing cells dramatically repressed IFNβ/IFNλ induction 5–10 fold versus WT or TTP KOs (Fig. 6A and B). The secretion of IFNβ was notably enhanced in ZIKV-infected TTP KO hBMECs 1–3 dpi, with TTP-expressing hBMECs repressing IFNβ secretion to levels 5- to 30-fold less than WT or TTP KO cells (Fig. 6C through E). A similar trend of IFNλ secretion 2–3 dpi in TTP KOs and reduced IFNλ secretion in TTP-expressing cells was observed but diminished (Fig. 6F through H). These findings reveal a novel mechanism of ZIKV-directed post-transcriptional IFN regulation directed by the induction of the ARE-BP TTP and demonstrates a novel role for ZIKV-induced TTP in regulating viral persistence and spread in endothelial cells.

### TTP KO does not alter IFN mRNA degradation

**FIG 7:**
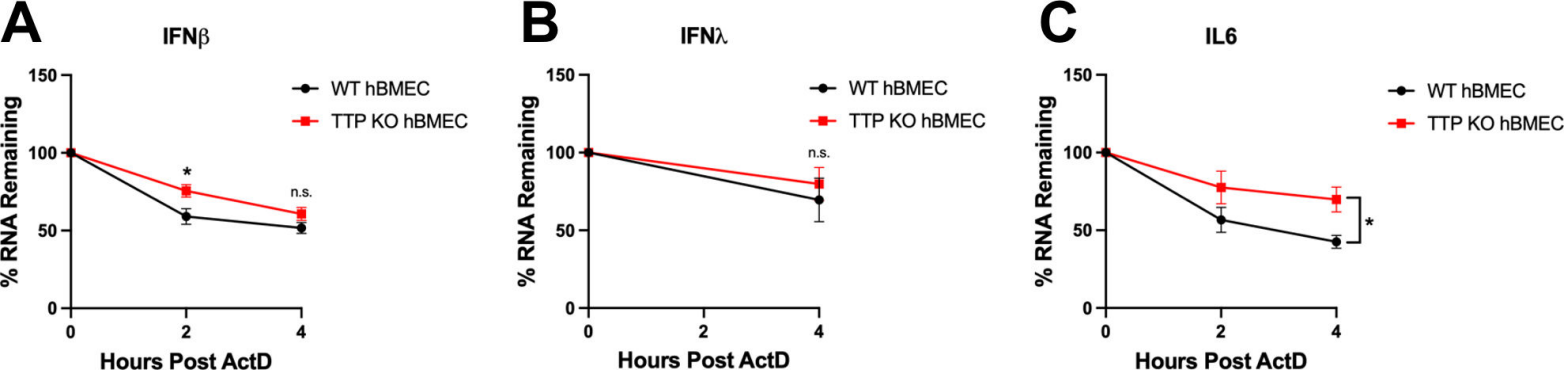
TTP KO does not alter IFN mRNA stability. Primary hBMECs and TTP-KO hBMECs were ZIKV infected (MOI = 10) for 20 h prior to the addition of actinomycin D (5 µg/mL). RNA was collected 0, 2, and 4 h post-ActD addition, and the levels of IFNβ (**A**), IFNλ_1_ (**B**), and IL-6 (**C**) mRNAs were analyzed via qRT-PCR and normalized to β-actin relative to the 0-h time point. Data are represented as the mean ± SEM; asterisks indicate statistical significance as determined by two-way ANOVA. *P ≤ 0.05. Experiments were performed three times. n.s., not significant.

In immune cells, TTP has been shown to post-transcriptionally repress cytokine expression through TTP complexes that translationally repress protein expression from ARE containing mRNAs or reduce the stability of ARE-mRNA transcripts. To differentiate TTP mechanisms of action, we evaluated the rate of IFNβ/IFNλ mRNA degradation in WT and TTP KO cells and compared the effect of TTP to the canonical TTP destabilizing target interleukin-6 (IL-6). WT hBMECs and TTP KO hBMECs were ZIKV infected (MOI = 10) and treated with actinomycin D (ActD) at 20 hpi to block new RNA transcription and were assayed for changes in IFNβ/IFNλ and IL-6 mRNA abundance (2–4 h post-ActD addition) by qRT-PCR. In TTP KO hBMECs, there was a noted reduction in the rate of IL-6 mRNA degradation that reflects the role of TTP in reducing IL-6 mRNA stability in immune cells (Fig. 7C) (69). In contrast, in ZIKV-infected hBMECs, TTP KO did not significantly alter the rate of IFNβ or IFNλ mRNA degradation compared to WT hBMECs (Fig. 7A and B). These findings are consistent with TTP inhibiting IFNβ/IFNλ secretion through the post-transcriptional inhibition of IFNβ/IFNλ mRNA translation in hBMECs.

### TTP regulates IFNβ/IFNλ expression in hSerCs

**Fig 8:**
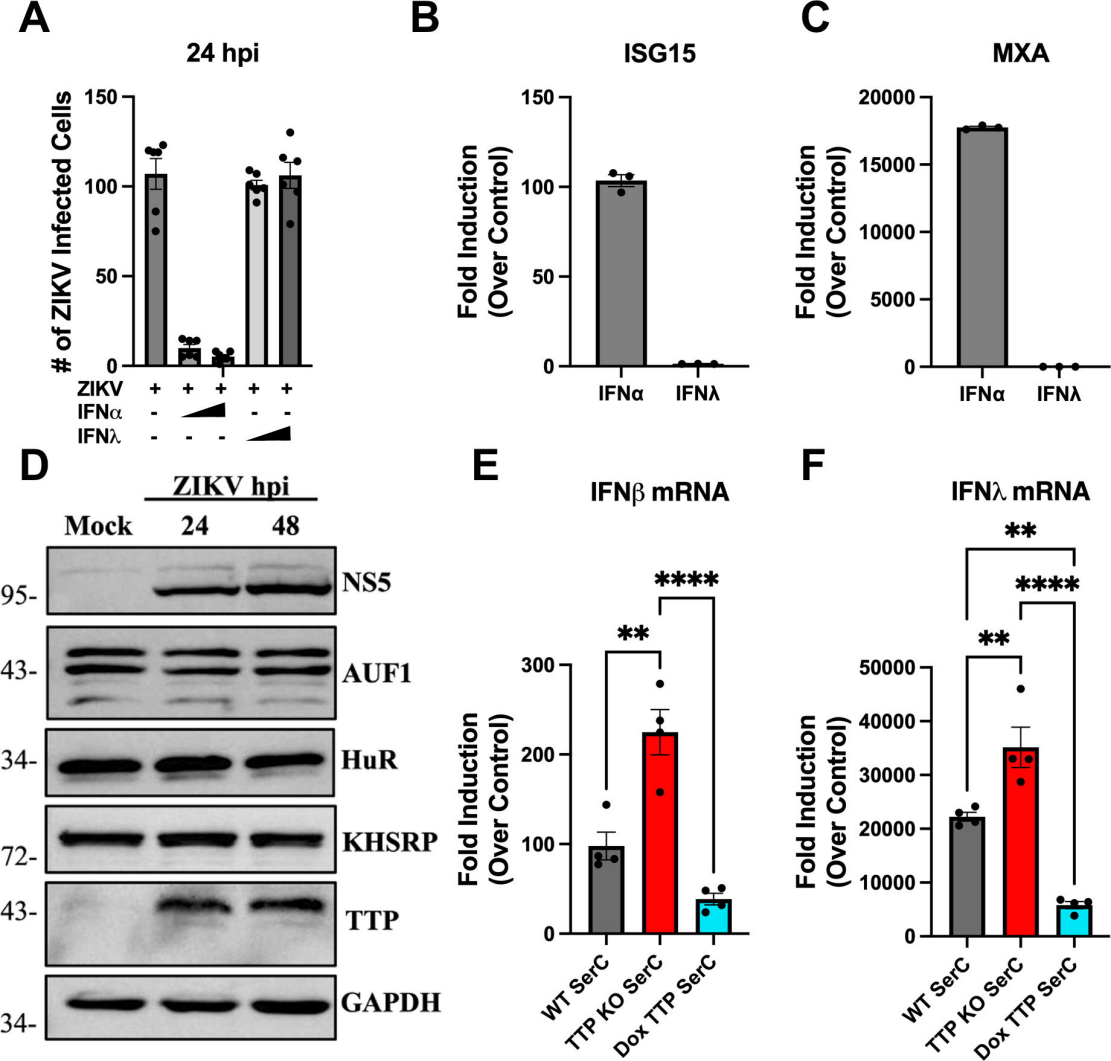
TTP regulates IFNβ/IFNλ expression in human Sertoli cells. (A through C) Primary hSerCs were ZIKV infected (MOI = 0.5) with or without IFNα (1,000 or 2,000 U/mL) or IFNλ_1_ (10 or 50 ng/mL) addition. ZIKV antigen-positive hSerCs were quantified for 24 hpi using anti-DENV4 hyperimmune mouse ascitic fluid (**A**), and RNA was collected to assess ISG induction relative to untreated, uninfected WT hSerCs (B and C). (**D**) Primary hSerCs were ZIKV infected (MOI = 10), and lysates were analyzed for ARE-BP expression by Western blot. (E and F) Representative Western blot from two experiments. ZIKV-infected (MOI = 10) primary hSerCs, TTP KO-hSerCs, and TTP-expressing hSerCs were assayed for IFNβ (**E**) and IFNλ (**F**) mRNAs for 24 hpi by qRT-PCR relative to WT, TTP KO, and Dox TTP uninfected control hSerCs. Data are presented as the mean ± SEM; asterisks indicate statistical significance as determined by one-way ANOVA. **P ≤ 0.002,****P ≤ 0.0001. Experiments were performed at least three times unless otherwise noted.

ZIKV persistently infects human Sertoli cells that protect testicular compartments, and viral persistence in this cellular niche is likely to enhance ZIKV sexual transmission (18, 19, 70). Sertoli cells produce both IFNβ and IFNλ and are sensitive to exogenous type I IFN treatment (Fig. 8A). Like hBMECs, the addition of IFNλ to Sertoli cells does not restrict ZIKV replication or induce ISGs (Fig. 8A through C, 1G and H). We initially determined if ZIKV infection directs ARE-BP responses that inhibit IFNβ/IFNλ secretion in hSerCs. Primary hSerCs were synchronously infected with ZIKV, and the expression of ARE-BPs was monitored 1–2 dpi. Like hBMECs, hSerCs showed no alteration in HuR, KHSRP, or AUF1 expression following ZIKV infection (Fig. 8D). In contrast, TTP expression was dramatically increased in ZIKV-infected Sertoli cells 1–2 dpi (Fig. 8D). To evaluate the role of TTP in regulating IFNβ/IFNλ mRNA levels in Sertoli cells, we generated TTP-KO and TTP-expressing hSerCs (Fig. S4). ZIKV-infected TTP KO hSerCs demonstrated a dramatic increase in IFNβ/IFNλ mRNA levels compared to ZIKV-infected WT controls (Fig. 8E and F). Conversely, ZIKV infection of TTP-expressing hSerCs reduced IFNβ/IFNλ mRNA levels by 60–85% when compared to WT or TTP-KO hSerCs (Fig. 8E and F). These findings demonstrate that ZIKV-induced TTP regulates IFNβ/IFNλ expression in Sertoli cells, key testicular barrier cells that serve as reservoirs for ZIKV dissemination and contribute to sexual transmission. ZIKV-directed TTP induction and IFN regulation reveal a fundamental ZIKV persistence mechanism that may prevent viral clearance from crucial physiological barriers.

## DISCUSSION

ZIKV is distinguished from other flaviviruses by its ability to persist in patients for up to 6 months, cause fetal microcephaly *in utero*, and be sexually transmitted (1 – 4, 71). To accomplish this, ZIKV persistently infects endothelial, trophoblast, and Sertoli cells that serve as barriers to immune protected brain, testicular, and placental compartments (12, 72 – 75). ZIKV persistence provides a reservoir for ZIKV dissemination and spread across tissue restrictive barriers (15). We previously reported that ZIKV persistently infects blood-brain barrier endothelial cells, spreading basolaterally from polarized hBMECs, a mechanism consistent with CNS entry. ZIKV persistence in hBMECs requires the autocrine activation of pro-survival CCL5 responses and the inhibition of IFNβ secretion (17). hBMECs secrete type I (IFNβ) and type III (IFNλ) IFNs; however, as hBMECs lack IFNλRs and fail to respond to IFNλ, ZIKV infection of hBMECs is only restricted by type I IFN pre-treatment. Despite transient induction of IFNβ/IFNλ in infected hBMECs, ZIKV-infected hBMECs failed to secrete detectable or functional IFNβ/λ and led us to hypothesize ZIKV post-transcriptionally inhibits IFNβ/IFNλ expression (15).

ZIKV impairs IFN induction and IFN receptor responses through multiple mechanisms. ZIKV NS3/NS5, additional NS proteins, and sfRNA reportedly suppress RIG-I/MDA-5 signaling pathway responses that induce IFN mRNA transcription (28 – 33). ZIKV also targets JAK/STAT signaling pathways downstream of IFNAR to restrict the induction of antiviral ISGs. NS2B3 degrades Jak kinase, preventing downstream STAT phosphorylation, while ZIKV NS5 inhibits STAT phosphorylation and promotes human STAT2 degradation (25, 28, 35, 36).

Despite ZIKV regulation of IFN induction, several reports note that IFN induction is dissociated from IFN secretion (9, 37, 39, 40). In ZIKV infected PBMCs IFN transcripts were induced, but PBMCs failed to secrete IFNα and IFNλ, and ZIKV-infected placental macrophages, fetal neural progenitors, and dendritic cells similarly failed to secrete IFNs (9, 37, 39, 40). In dendritic cells, a comparison of IFN cDNA synthesis (hexamer versus oligo-dT) suggested IFN mRNA stability was unchanged by ZIKV infection, and translational IFN regulation was inferred (37). Collectively, IFN induction without IFN secretion following ZIKV infection was noted in immune cells, where TTP expression in response to LPS is well characterized, and mirrored our own observations of ZIKV inhibiting IFN secretion in hBMECs at a translational level (9, 37, 39, 40).

ZIKV infection reportedly induces the expression of miRNAs that have been shown to regulate the production of ISGs, cell death, and chemotaxis and represent a potential mechanism of post-transcriptional IFN regulation (76 – 78). Although ZIKV induction of miRNAs targeting IFNβ/IFNλ has not been reported, the distantly related *Flavivirus* hepatitis C virus (HCV) induces miRNAs targeting IFNλ to restrain IFN signaling and promote viral persistence in hepatocytes (79, 80). We initially assessed miRNAs induced in ZIKV-infected hBMECs at 24 hpi but found no significant upregulation of canonical IFN targeting miRNAs (mir-34a, mir-145, or let-7b), with the IFNβ targeting miRNA, miR-34a, induced by only 1.61-fold (*P* value = 0.06) (81).

In HCV pathogenesis, IFNλ ARE-mediated inhibition has been implicated. In the 3′ UTR of *IFNL3*, an ARE polymorphism was identified that interfered with ARE sequence recognition, ultimately enhancing IFNλ_3_ production and conferring cellular resistance to HCV (80, 82, 83). Both *IFNB1* and *IFNL1* transcripts produced by ZIKV-infected hBMECs contain AREs in their 3′ UTRs (48, 84). This led us to hypothesize the AREs in IFNβ/IFNλ mRNAs as targets of ZIKV post-transcriptional regulation and potential roles for ARE-BPs in ZIKV persistence in hBMECs. During ZIKV infection, ARE-BPs have primarily been observed as markers of stress granule localization but ARE-BPs as determinants of spread and persistence have remained understudied (85 – 87). Knockdown of HuR, an ARE-BP that stabilizes IFN mRNAs, was shown to increase ZIKV titers, although the effect on IFN expression was not assessed (42, 87).

Due to reports of ARE-BPs regulating IFN mRNAs, we assessed the expression and localization of TTP, KHSRP, AUF1, and HuR in ZIKV-infected hBMECs (41). We found that only TTP was induced by ZIKV infection and that TTP was localized to the cytoplasm. Importantly, ZIKV-directed TTP expression was independent of IRF3-directed IFN induction, and TTP was not induced in response to IFN addition to cells. How ZIKV triggers TTP induction independent of IRF3 is unclear and warrants further study but likely involves regulation by p38 MAPK. ZIKV infection reportedly activates p38 MAPK, a key regulator of LPS-directed TTP induction, activity, and protein stability (52, 62, 88, 89).

IFN regulation remains an understudied aspect of TTP biology and has not been addressed in the context of viral infections. Impaired IAV replication following TTP knockdown was attributed to an increase in COX-2 protein; however, the effect of TTP on IFN production was not assessed (67). Two studies have implicated IFNβ and IFNγ as targets of TTP repression in LPS-treated murine macrophages and T cells, respectively; however, TTP regulation of IFN has not been assessed in virally infected, non-immune human cells (90, 91). We observed that TTP expression in hBMECs and Sertoli cells regulates IFNβ/IFNλ mRNA abundance and protein expression in response to ZIKV infection. These cells comprise critical physiological barriers protecting brain and testicular tissues, respectively, and the ability to limit IFN responses likely fosters viral persistence and spread into these immune privileged compartments.

Apart from this study, the role of TTP in regulating IFN responses that restrict viral infection and spread in human cells has yet to be investigated, but TTP expression in a variety of cell types underscores its potential importance. In addition to TTP expression in brain, testicular, placental, and ovarian tissues, TTP is the most highly expressed in cervical tissue (92). IFNλ secretion protects the female reproductive track and plays a critical role in preventing viral spread across placental barriers (93, 94). TTP expression by placental cells, including Hofbauer cells, has not been investigated; however, TTP expression may suppress IFN production by key mediators of ZIKV persistence to facilitate viral spread and congenital fetal infection. In these barrier settings, TTP-regulated IFNβ/IFNλ responses are likely to be critical to ZIKV sexual transmission, persistence, and *in utero* spread to fetal tissues.

To determine if IFNβ/IFNλ mRNA and secreted protein levels are altered due to TTP destabilization of IFN mRNAs, we assessed IFNβ, IFNλ, and IL-6 degradation rates following addition of actinomycin D. The ability of TTP to promote IL-6 mRNA degradation is well characterized in immune cells in response to LPS (69, 95). However, TTP regulation of IFNs is less clear with TTP overexpression enhancing IFNβ mRNA degradation 1 h, but not 4 h, post-LPS treatment, in murine bone marrow-derived macrophages (BMDMs) (91). In contrast, KO of a TTP activator, DUSP1, stabilized IFNβ mRNA only at 4 h post-LPS treatment, indicating that TTP interactions with IFNβ are temporally regulated (91).

TTP’s ability to promote mRNA degradation is dependent on a complex and dynamic balance of phosphorylation and dephosphorylation events downstream of the MAPK signaling cascade and is largely mediated by p38 MAPK, MK2, DUSP1, and PP2A (60, 96). More investigation is required to fully characterize the phosphorylation status and expression of these TTP regulating proteins to determine how they influence TTP activity and transcript interaction during ZIKV infection. In TTP KO hBMECs, we observed no significant alteration to IFNβ/IFNλ mRNA stability but did detect faster degradation of IL-6 mRNA. We assessed IFN mRNA stability at 24 hpi because it coincides with the peak of IFN induction and TTP expression following a synchronous ZIKV infection of hBMECs. However, we cannot discount altered IFN mRNA stability at other times post-infection, and it is unclear how IFN mRNA levels are enhanced during ZIKV infection of TTP KO cells. TTP also reportedly functions as a transcriptional co-repressor, inhibiting TNF-α promotor activity through interactions with NF-κB (60). TTP transcriptional repression of IFNs has not been investigated; however, we consider this regulation to be unlikely as it requires nuclear localization of TTP, which we did not observe during ZIKV infection of hBMECs (Fig. 2B). Ultimately, our findings are consistent with ZIKV-induced TTP repressing the translation of IFNβ/IFNλ mRNAs to suppress IFN secretion.

TTP has multiple mechanisms of repressing ARE-mRNA translation and has been shown to directly form complexes with poly(A)-binding protein and the inhibitory eukaryotic initiation factor 4E2 (60, 61, 97). Recruitment of eIF4E2 to ARE-mRNAs prevented their association with eIF4E, a scaffold protein required for assembly of the translation initiation complex leading to the inhibition of cap-dependent translation (60, 61, 97). TTP also reportedly interacts with GYF2 to recruit the cap-binding translational repression complex 4EHP that competes for cap binding with eIF4E (59). However, the formation of translation regulating TTP complexes with IFN mRNAs and has not been addressed in endothelial cells, Sertoli cells, or trophoblasts that normally protect tissues from viral access (60, 61, 97).

Overall, our findings characterize a novel role for TTP in suppressing IFNβ/IFNλ production in primary human BMECs and Sertoli cells in response to ZIKV infection. We found that TTP is induced by ZIKV independent of IRF3 and IFN activity, and that TTP post-transcriptionally inhibits IFNβ/IFNλ protein expression and secretion despite transcriptional induction. These novel findings demonstrate that TTP regulation of IFN responses contributes to ZIKV spread and persistence and provides an additional mechanism for induced TTP to regulate IFNλ expression, a critical determinant of ZIKV placental transmission (98). Our findings underscore the complexity of the IFN response to ZIKV and highlight a new post-transcriptional mechanism of innate immune interference that prevents IFN secretion and fosters ZIKV persistence and spread across key tissue barriers.

## MATERIALS AND METHODS

### Cells

C6/36 cells (ATCC CRL-1660) were grown in Dulbecco’s modified Eagle’s medium (DMEM) supplemented with 10% fetal bovine serum (FBS), penicillin (100 µg/mL), streptomycin sulfate (100 µg/mL), and amphotericin B (50 µg/mL, Mediatech) at 28°C with 5% CO_2_. Human brain microvascular endothelial cells were purchased from Cell Biologics (H-6023) and grown in endothelial cell growth basal medium-2 with SingleQuots (Lonza) at 37°C in 5% CO_2_. hBMECs were discarded upon reaching passage 12. Human Sertoli cells were purchased from ScienCell (#4520) and grown as above in Sertoli cell media (#4521). Vero E6 (ATCC CRL 1586), HEK293T (ATCC), and A549 (ATCC CCL-185) were grown in DMEM as above with 8% FBS.

### Virus

ZIKV (PRVABC59) was obtained from the ATCC (VR-1843) and passaged in C6/36 cells according to the manufacturer’s instructions. Infectious ZIKV stocks (5 × 10^5^ FFU/mL) were generated by inoculating confluent T75 flasks of C6/36 cells for 4 days in 2% DMEM before transfer to T175 flasks for 6 days prior to harvest and centrifugation of viral stock. Viral titers were determined by serial dilution and quantification of infected foci in Vero E6 cells for 24 hpi via immunoperoxidase staining with anti-DENV4 hyperimmune mouse ascitic fluid (ATCC), horseradish peroxidase-labeled anti-mouse IgG (1:2,000; KPL-074-1806), and 3-amino-9-ethylcarbazole. ZIKV infections were performed by absorbing virus onto primary hBMEC/hSerC monolayers (2–4 × 10^5^ cells) for 2 h at 37°C, washing with phosphate-buffered saline (PBS), and resupplementing with media. For all experiments involving doxycycline induced, TTP-expressing hBMECs and hSerCs, 200-ng/mL doxycycline was maintained in culture media throughout the course of ZIKV infection.

### Induction and expression analysis

hBMECs (2 × 10^5^) were ZIKV infected, transfected with 0.5-µg/mL poly(I:C) and FuGENE 6 (1:3) or treated with 1,000 U/mL IFNα (Sigma) for 24 h prior to supernatant and RNA collection. For IFN and ISG induction experiments, IFNα (Sigma) and IFNλ_1_ generated from IFNλ_1_-expressing HEK-293T cells were added to hBMEC/hSerCs/A549 concurrently with ZIKV (MOI = 0.5). Following inoculum removal, indicated concentrations of IFNα/IFNλ_1_ were added back to monolayers before cell fixation or RNA collection for 24 hpi.

### Lentivirus, transduction, and selection

pLentiCRISPRv2 plasmid was purchased from Addgene (#52961), and gRNA was inserted as described previously (99, 100). pLV2CRISPR-hCas9:T2A: Puro-U6 was purchased from VectorBuilder for TTP KO. Lentivirus was produced in HEK 293 T cells following PEI transfection (5 µg/1 µg DNA) of pLentiCRISPRv2, psPAX2 (Addgene #12260), and pLp/VSVG (Invitrogen) and was collected 5 days post-transfection. hBMECs were doubly transduced over 48 h and selected with puromycin (0.8 µg/mL) for 72 h prior to KO validation by Western blot. gRNAs used can be found in Table S1.

pCW57-MCS1-2A-MCS2 was purchased from Addgene (#71782) and used to conditionally express TTP and IFNλ_1_ derived from ZIKV-infected hBMEC cDNA in the presence of doxycycline (0.2–1.0 µg/mL). TTP and IFNλ_1_ were cloned using primers found in Table S1 and inserted between pCW57-MCS1-2A-MCS2 EcoRI (NEB #R3101S) and BamHI (NEB #R3136S) restriction sites.

### qRT-PCR analysis

RNA was purified using RNeasy Mini Kit (Qiagen) according to the manufacturer’s instructions. cDNA was generated using Transcriptor first-strand cDNA synthesis kit (Roche) and ProtoScript II first-strand cDNA synthesis kit (New England BioLabs) using Oligo-p(dT)_15_ or d(T)_23_ VN primers. qRT-PCR primers used are found in Table S1.

cDNA was amplified using PerfeCTa SYBR Green SuperMix (QuantaBio) on a BioRad CFX96 Touch Real-Time PCR Detection System. All genes were normalized to internal β-actin controls, and gene induction was determined using 2^−ΔΔCt^ over uninfected/uninduced controls of the same cell type. For mRNA half-life determination, hBMECs were treated with 5-µg/mL actinomycin D (Sigma) for the indicated times prior to RNA purification. mRNA abundance over time was plotted relative to RNA collected immediately following actinomycin addition and was assessed for significance via one-way analysis of variance using GraphPad Prism.

### ELISA

Levels of secreted IFNβ and IFNλ in mock, ZIKV-infected, and poly (I:C)-treated supernatants were determined using DuoSet ELISA (R&D Systems) according to the manufacturer’s instructions. ELISA plates (Immunolon 2, U-bottom; Dynatech Laboratories) were developed using tetramethylbenzidine, and optical density (O.D.) was measured using a Molecular Devices SpectraMax M5 plate reader (450 nM). Protein concentrations were calculated by fitting O.D. to a standard curve of purified IFN using SoftMax Pro software.

### Western blot analysis

Cells were washed in PBS prior to lysis on ice in buffer containing 1% NP-40, 0.1% SDS, 150 mM NaCl, 50 mM Tris-HCl pH 7.4, 2 mM EDTA, 10 nM NaF, 1 mM phenylmethylsulfonyl fluoride (PMSF), and protease inhibitor cocktail (Sigma). Protein concentration was determined using Pierce BCA Protein Assay Kit (Thermo) and 36 µg of protein was resolved on a 10–12% SDS polyacrylamide gel. Proteins were transferred to a 0.4- µM nitrocellulose membrane and blocked in either 5% bovine serum albumin or 5% non-fat milk prior to incubation with primary antibody. Cellular fractionation was performed as previously described (101). Antibodies used include ZIKV NS5 1:1,000 (GeneTex #GTX133327), AUF1 1:1,000 (Millipore 07–260), lamin B1 1:1,000 (Cell Signaling D4Q4Z), TTP 1:250 (Cell Signaling D1I3T), IRF3 1:1,000 (Cell Signaling D83B9), Myc 1:1,000 (Cell Signaling 2272S), KHSRP 1:1,000 (Cell Signaling E2E2U), HuR 1:1,000 (Santa Cruz sc-5261), and GAPDH 1:3,000 (Sigma G9545).

### Statistical analysis

Results in each figure were derived from data collected from ≥3 independent experiments and were displayed as the mean ± standard error of the mean. One-way analysis of variance, two-way analysis of variance, and unpaired Student’s *t* test were used where indicated, with *P* values of ≤0.05 considered significant. All statistical tests were performed using GraphPad Prism version 9.

## SUPPLEMENTAL MATERIAL

Supplemental Figure Legends mbio.01742-23-s0001.docx Legends to Table S1 and Fig. S1 to 4.

Figure S1 mbio.01742-23-s0002.tif IFNα/λ induce ISGs in A549 cells.

Figure S2 mbio.01742-23-s0003.tif ZIKV induction of MXA, IFIT1, and CCL5 is IRF3 dependent.

Figure S3 mbio.01742-23-s0004.tif Validation of hBMEC TTP KO and doxycycline-induced TTP expression.

Figure S4 mbio.01742-23-s0005.tif Validation of hSerC TTP KO and doxycycline-induced TTP expression.

Table S1 mbio.01742-23-s0006.tif Oligonucleotide primers.

ASM does not own the copyrights to Supplemental Material that may be linked to, or accessed through, an article. The authors have granted ASM a non-exclusive, world-wide license to publish the Supplemental Material files. Please contact the corresponding author directly for reuse.
