# Supplementary material for: ZIKV induction of tristetraprolin in endothelial and Sertoli cells post-transcriptionally inhibits IFNβ/λ expression and promotes ZIKV persistence
Source: mBio. 2023 Sep 14;14(5):e01742-23. doi: 10.1128/mbio.01742-23 (PMC10653947; doi:10.1128/mbio.01742-23)
Supplement: Supplemental Figure Legends — Legends to Table S1 and Fig. S1 to 4. [file mbio.01742-23-s0001.docx]

**Supplementary Table and Figures**

**Table S1: List of primers.** Primers referenced in the methods are detailed.

**Figure S1: IFNα/λ Induce ISGs in A549 Cells.** (A, B) A549 cells were treated with 2000 U/mL IFNα or 50 ng/mL IFNλ for 24 hours prior to collection of RNA. qRT-PCR was performed to assess the induction of representative ISGs, ISG15 (A) and MXA (B) relative to untreated control cells. IFN treated cells were normalized to untreated, mock-infected A549s.

**Figure S2:** **ZIKV Induction of MXA, IFIT1, and CCL5 is IRF3 Dependent.** WT hBMECs and hBMECs expressing dominant negative IRF3Δ60 were ZIKV infected (MOI 1) for indicated times prior to qRT-PCR analysis of MXA (A), IFIT1 (B), and CCL5 (C) relative to uninfected WT and IRF3Δ60-hBMECs. Data are presented as the mean ± SEM. Experiments were performed 3 times.

**Figure S3: Validation of hBMEC TTP KO and Doxycycline-Induced TTP Expression.** (A, B) WT and TTP KO hBMECs were ZIKV infected for 24 hours (MOI 1). The KO of TTP (A) and expression of TTP (B) confirmed by Western blot.

**Figure S4: Validation of hSerC TTP KO and Doxycycline-Induced TTP Expression.** WT and TTP KO hSerCs were ZIKV infected for 24 hours (MOI 1) and the KO of TTP confirmed by Western blot (A). WT hSerCs were ZIKV infected for 24 hours (MOI 1) and TTP expression compared by western blot to Dox-TTP hSerCs with and without doxycycline stimulation (B).
