# Supplementary figures and images for: ZIKV induction of tristetraprolin in endothelial and Sertoli cells post-transcriptionally inhibits IFNβ/λ expression and promotes ZIKV persistence
Source: mBio. 2023 Sep 14;14(5):e01742-23. doi: 10.1128/mbio.01742-23 (PMC10653947; doi:10.1128/mbio.01742-23)

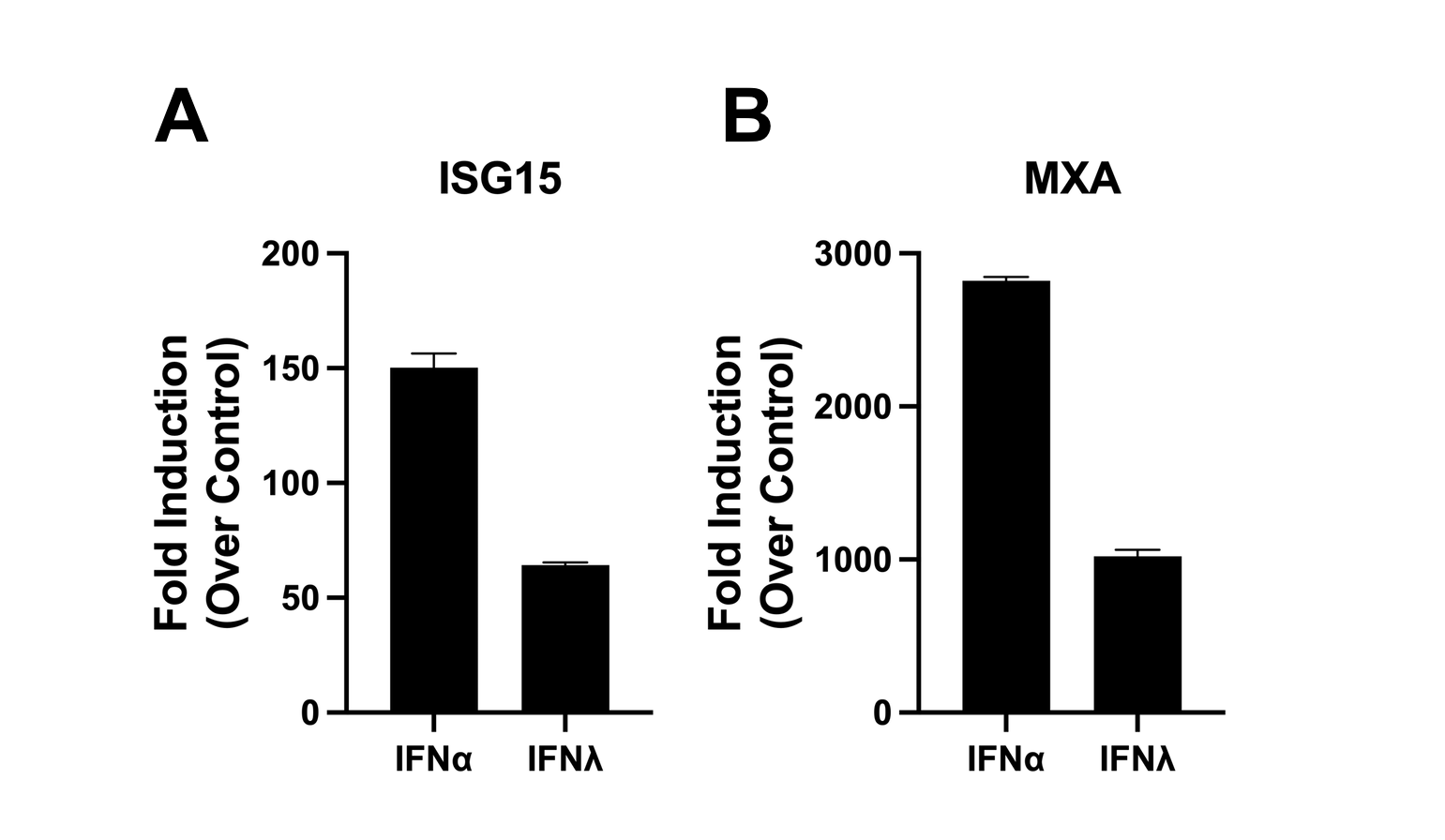

Supplement: Figure S1 — IFNα/λ induce ISGs in A549 cells. [file mbio.01742-23-s0002.tif]

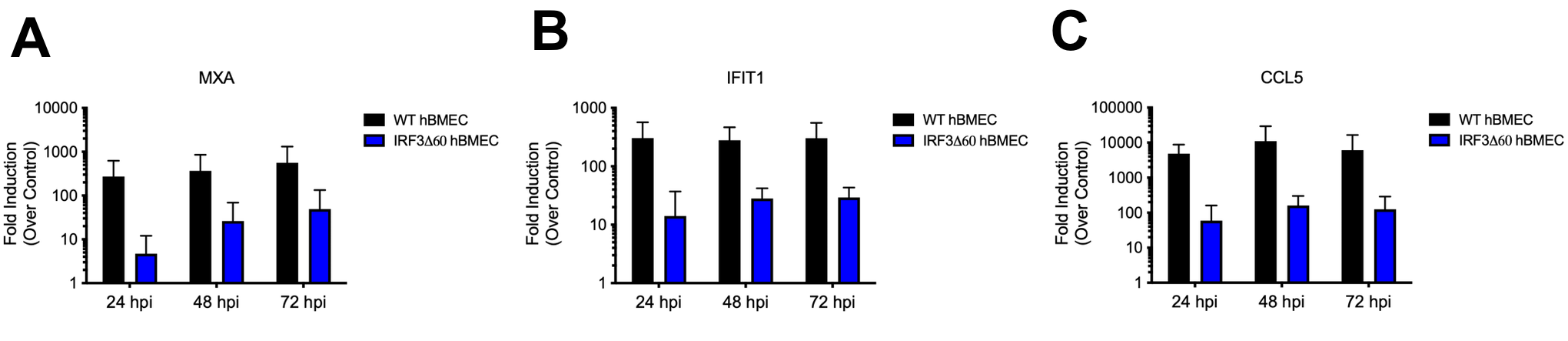

Supplement: Figure S2 — ZIKV induction of MXA, IFIT1, and CCL5 is IRF3 dependent. [file mbio.01742-23-s0003.tif]

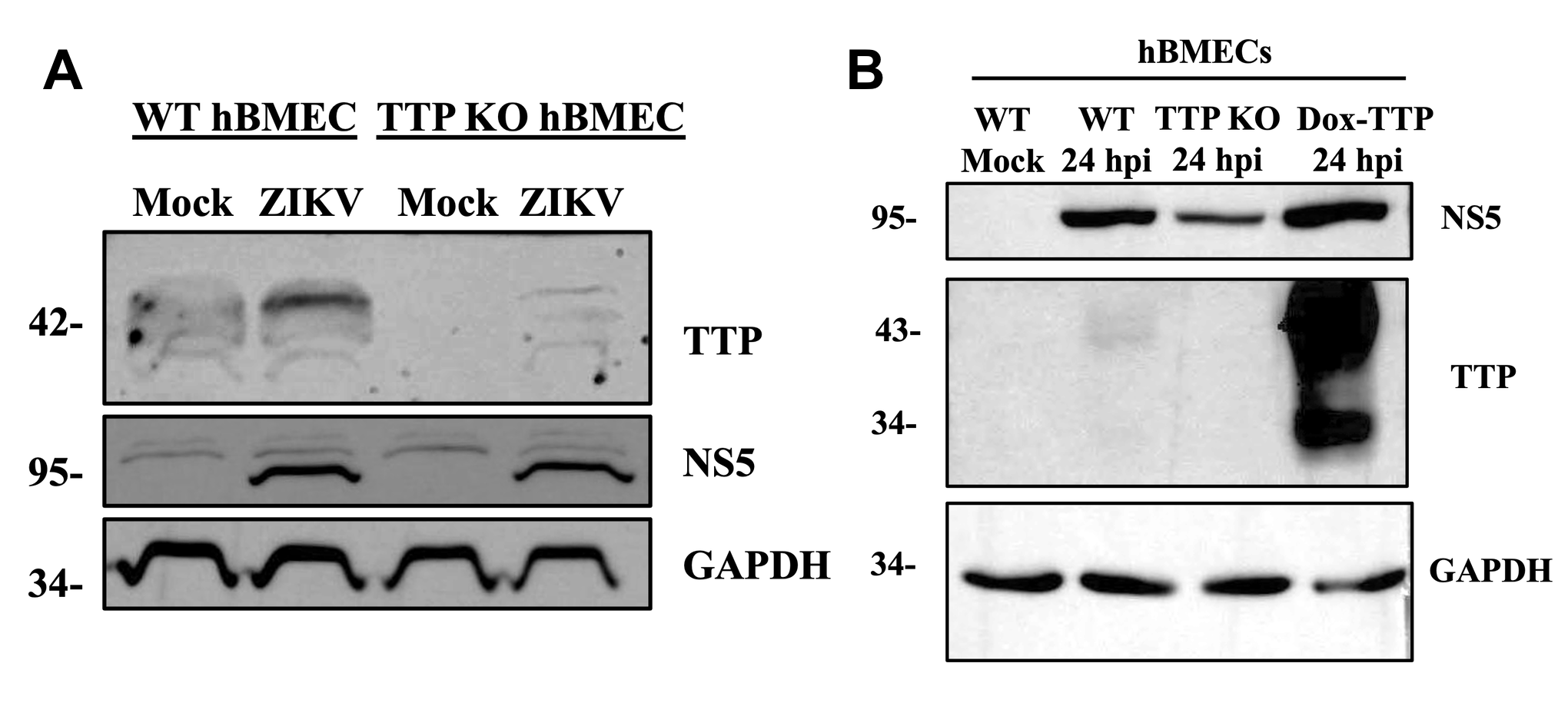

Supplement: Figure S3 — Validation of hBMEC TTP KO and doxycycline-induced TTP expression. [file mbio.01742-23-s0004.tif]

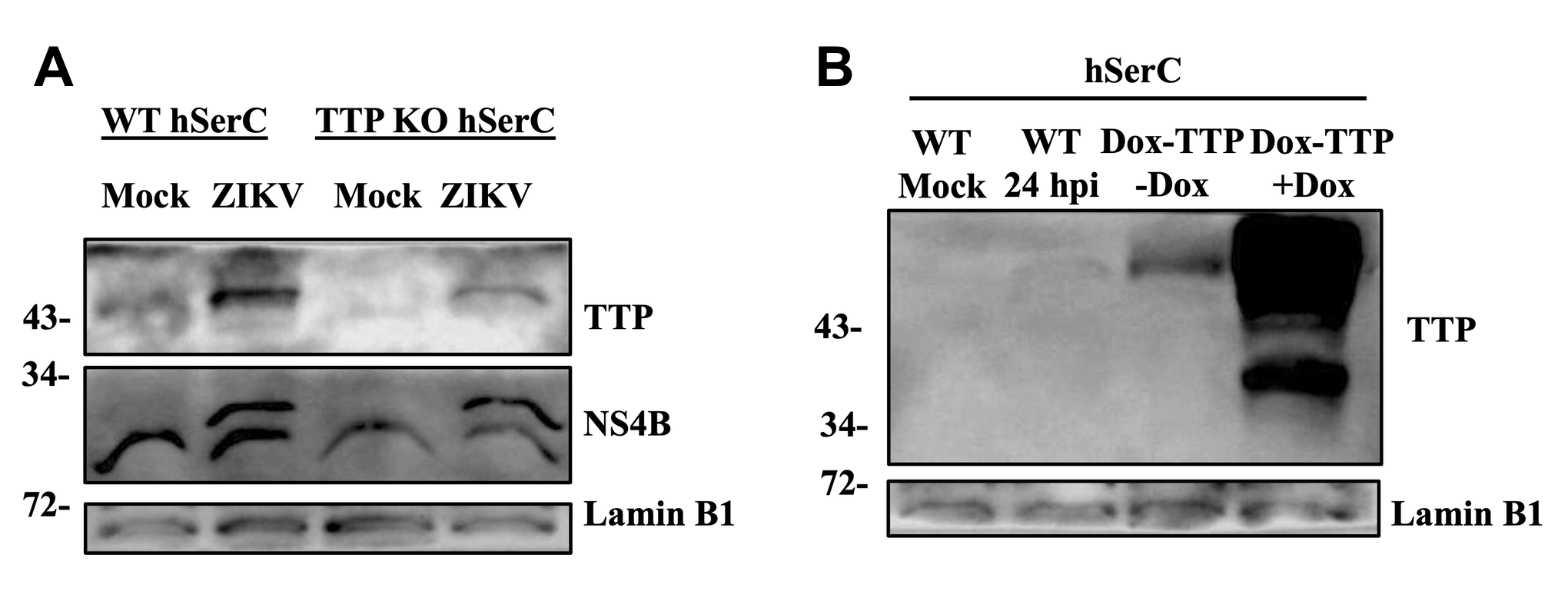

Supplement: Figure S4 — Validation of hSerC TTP KO and doxycycline-induced TTP expression. [file mbio.01742-23-s0005.tif]

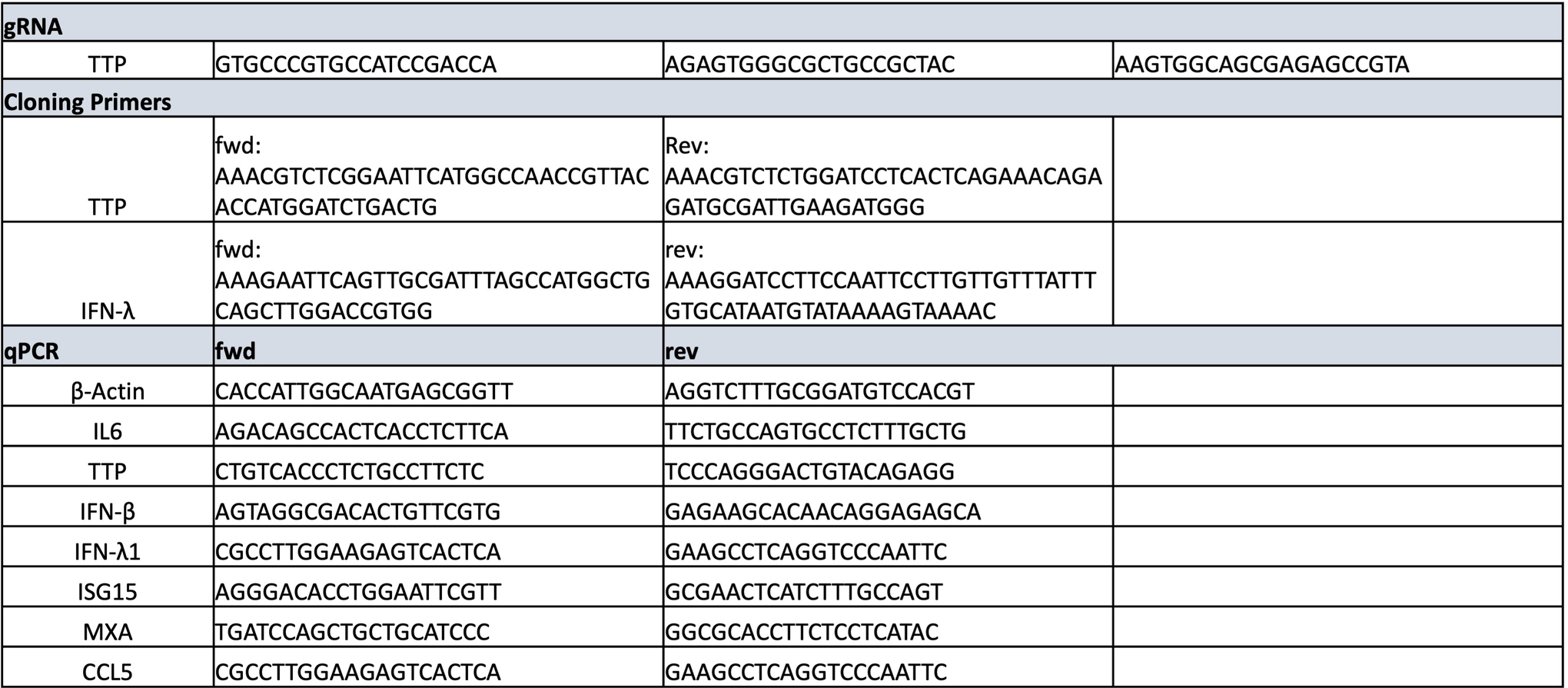

Supplement: Table S1 — Oligonucleotide primers. [file mbio.01742-23-s0006.tif]
